# Design of Innovative Biocompatible Cellulose Nanostructures for the Delivery and Sustained Release of Curcumin

**DOI:** 10.3390/pharmaceutics15030981

**Published:** 2023-03-18

**Authors:** Francisca Casanova, Carla F. Pereira, Alessandra B. Ribeiro, Eduardo M. Costa, Ricardo Freixo, Pedro M. Castro, João C. Fernandes, Manuela Pintado, Óscar L. Ramos

**Affiliations:** CBQF—Centro de Biotecnologia e Química Fina–Laboratório Associado, Escola Superior de Biotecnologia, Universidade Católica Portuguesa, Rua Diogo Botelho 1327, 4169-005 Porto, Portugal

**Keywords:** nanocellulose, curcumin, delivery systems, sustained release, cytotoxicity

## Abstract

Poor aqueous solubility, stability and bioavailability of interesting bioactive compounds is a challenge in the development of bioactive formulations. Cellulose nanostructures are promising and sustainable carriers with unique features that may be used in enabling delivery strategies. In this work, cellulose nanocrystals (CNC) and cellulose nanofibers were investigated as carriers for the delivery of curcumin, a model liposoluble compound. Nanocellulose modification with the surfactant cetyltrimethylammonium bromide (CTAB), tannic acid and decylamine (TADA), and by TEMPO-mediated oxidation were also tested and compared. The carrier materials were characterized in terms of structural properties and surface charge, while the delivery systems were evaluated for their encapsulation and release properties. The release profile was assessed in conditions that mimic the gastric and intestinal fluids, and cytotoxicity studies were performed in intestinal cells to confirm safe application. Modification with CTAB and TADA resulted in high curcumin encapsulation efficiencies of 90 and 99%, respectively. While no curcumin was released from TADA-modified nanocellulose in simulated gastrointestinal conditions, CNC-CTAB allowed for a curcumin-sustained release of ca. 50% over 8 h. Furthermore, the CNC-CTAB delivery system showed no cytotoxic effects on Caco-2 intestinal cells up to 0.125 g/L, meaning that up to this concentration the system is safe to use. Overall, the use of the delivery systems allowed for the reduction in the cytotoxicity associated with higher curcumin concentrations, highlighting the potential of nanocellulose encapsulation systems.

## 1. Introduction

Poor bioavailability of bioactive compounds is an increasingly pronounced challenge in the development of bioactive formulations. Around 40% of marketed active compounds and up to 70% of candidates showing high potential in the pipeline of pharmaceutical, nutraceutical and food industries show hydrophobicity, liposolubility or poor aqueous solubility, resulting in limited potential and unsatisfactory efficacy when administrated orally [1,2,3]. Bioactive compounds such as lipophilic phenols, carotenoids, lipophilic vitamins or phytosterols have very interesting biological functions, but their low water solubility and stability result in poor bioavailability, restricting their applications [4,5]. These limitations can potentially be overcome by using enabling delivery systems, which require carrier materials with desirable properties [6,7]. Certain liposoluble compounds have been used as model bioactive molecules to study encapsulation strategies, amongst which special interest has been given to curcumin, a natural phenolic compound with important biological properties, namely antimicrobial, anti-inflammatory, anti-mutagenic and cholesterol-lowering activities [8,9,10,11].

Nowadays, there is a growing need for the utilisation of biocompatible raw materials for the delivery of liposoluble compounds [12]. Proteins, e.g., whey protein [13], caseins [14], gelatin [15] and soy proteins [16] are biocompatible carrier materials, but they tend to aggregate and are susceptible to disruption under physiological conditions in the gastrointestinal (GI) tract [17]. Lipid-based carriers, such as lipossomes [18], emulsions [19], nano-structured lipid carriers [20] and solid lipid nano-particles [21] are safe and attractive carriers; however, these materials might experience undesirable phenomena (e.g., Ostwald ripening, aggregation, oxidation, gelation) resulting from their physical and chemical instability. Considerable attention has been drawn to natural polysaccharides, namely chitosan [22], cyclodextrins [23], amylose [24], alginate [25], starches [26], pectin [27] and cellulose [4,28], due to their abundance, low cost, low toxicity, biocompatibility and biodegradability. Cellulose is the world’s most abundant natural polymer and it can be obtained from several renewable and sustainable plant sources, such as lignocellulosic biomass from industrial and agricultural wastes. Non-plant sources of cellulose also exist, e.g., cellulose produced by bacteria, algae and tunicates [29,30,31]. The native cellulose fiber structure is composed of nanostructured cellulose (NC), which can be further divided into cellulose nanofibers (CNF) and cellulose nanocrystals (CNC). These have essentially different extraction procedures, as well as different dimensions, morphologies and crystalline structures, as reviewed by Casanova et al. (2021) [11]. Still, both CNF and CNC are composed of repeating β-D-glucopyranose units with three hydroxyl groups per anhydroglucose unit (AGU). Interest in cellulose nanostructures as carriers in delivery systems has increased in the past few years due to their unique physicochemical properties, such as renewability, biocompatibility, biodegradability, high surface area and amphiphilic nature [11].

Nevertheless, surface modification of cellulose structures may be necessary in order to optimize the loading and release profile of encapsulated lipophilic compounds such as curcumin, as ascertained by Zainuddin et al. (2017) and Foo et al. (2019) [4,11,28]. In fact, one interesting cellulose feature is the surface chemical reactivity and accessibility of hydroxyl groups for chemical modification, which may provide additional functionalities to the cellulose molecule [32]. Hydrophobic surface modification [4,28] and functionalization of cellulose structures by TEMPO-mediated oxidation (2,2,6,6-tetramethylpiperidine 1-oxyl–TEMPO) [33] have been employed to modulate the encapsulation and delivery of lipophilic compounds, namely curcumin and carvacrol. Hydrophobic modification with cetyltrimethylammonium bromide (CTAB, a hydrophobic cationic type surfactant) [28,34,35], and with tannic acid (TA) and decylamine (DA) [4] have been previously studied for the encapsulation of lipophilic molecules showing high encapsulation efficiencies superior to 90%. However, the biocompatibility of such delivery systems has rarely been analyzed, and this information is of crucial importance for the applicability of the systems (considering that surfactants and amino compounds are being used and are possibly toxic). Furthermore, studies showing the release profile of encapsulated bioactive compounds from such delivery systems are also scarce.

In the present work, CNC and CNF are investigated as delivery systems for curcumin, a model liposoluble compound. Hydrophobic modification with CTAB and TADA, and functionalization by TEMPO-mediated oxidation, were also tested and compared, for both CNC and CNF, in order to evaluate the influence of the different approaches in the encapsulation and release of curcumin. This work exploited the physicochemical and encapsulation properties of such cellulose-based delivery systems, but also the release profile of the model lipophilic compound (curcumin) in conditions that mimic the gastrointestinal fluids (pH, temperature and time of exposure). Moreover, the biocompatibility of the developed delivery systems through in vitro cytotoxicity studies was also assessed. To the best of our knowledge, this study is unique as there are no reported studies comparing the performance of different approaches (including chemical modified materials) simultaneously and for both CNC and CNF intended for the encapsulation of lipophilic compounds. Additionally, the release profile under gastrointestinal conditions of such systems has been investigated to a far lesser extent, and biocompatibility and toxicity studies, which are vital to assess the applicability of said systems, are rarely performed.

## 2. Materials and Methods

### 2.1. Reagents

The reagents used in the experiments were of analytical grade or higher. Commercial CNC, CNF and CNF-TEMPO (1.4 mmol COONa/g) were kindly supplied by Cellulose Lab (Fredericton, Canada). According to the supplier, CNC had a needle-like morphology with 10–20 nm width and 50–400 nm length; CNF had a fibrillar morphology with a fiber width of 50 nm and lengths of up to several hundred microns; CNF-TEMPO had a fibrillar morphology with a fiber width of 40 nm and lengths of up to several hundred microns. CTAB (cetyltrimethylammonium bromide) (>99.0%), tannic acid (>99.0%), potassium phosphate (>99.0%), hydrochloric acid (32%) and dimethyl sulfoxide (>99.9%) were purchased from Sigma-Aldrich (St. Louis, MO, USA). Decylamine (99.0%) was purchased from Thermo Fisher Scientific (Waltham, MA, USA), sodium hydroxide (>95.0%) and sodium chloride (>99.5%) were purchased from Labchem (Zelienople, PA, USA) and ethanol (>99.8%) was purchased from Honeywell (Charlotte, NC, USA). 

### 2.2. Production of CNC-TEMPO

CNC-TEMPO was produced from commercial CNF-TEMPO by a method adapted from Zhou et al. (2018) [36]. Briefly, a CNF-TEMPO suspension (0.5% *w*/*v*) was subjected to ultrasonic irradiation for 10 min with a JP Selecta CY-500 Ultrasonic Homogenizer (Barcelona, Spain) in an ice bath to avoid overheating. The processor was equipped with a cylindrical titanium alloy probe tip of 10 mm diameter. Sonication was performed at 80% amplitude for 5 min at a time to replace the ice bath (500 W, 20 kHz, pulse durations of 15 s on and 5 s off). Aqueous suspension of CNC-TEMPO was freeze-dried for further use. CNC-TEMPO was produced with a 96.8% ± 1.73 yield from commercial CNF-TEMPO.

### 2.3. CNC and CNF Modification with CTAB Surfactant

Commercial CNF and CNC (0.4%, *w*/*v* aqueous suspension) were modified with an aqueous solution of CTAB (2.0 mM) by a method adapted from Zainuddin et al. (2017) [28]. The nanocellulose suspensions were slowly added into the CTAB solution, and the mixture was heated at 60 °C for 3 h. The reaction was left stirring at room temperature overnight. The unbound CTAB was removed by centrifugation at 10,000 rpm for 10 min. The pellet was washed with distilled water, followed by another round of centrifugation. This step was repeated twice. Aqueous suspensions of CNF-CTAB and CNC-CTAB were freeze-dried for further use and demonstrated production yields of 87.95% ± 2.01 and 82.45% ± 1.26 from CNF and CNC, respectively. The degree of substitutions (DS) was measured by the percentage of nitrogen (% N) according to Zainuddin et al. (2017) using the Dumas method in a Foss Dumatec™ 8000 (Hillerød, Denmark) [28,37]. CNF-CTAB had a substitution degree (DS) of 0.081 ± 0.011 and CNC-CTAB a DS of 0.124 ± 0.005. 

### 2.4. CNC and CNF Modification with TA and DA 

The method of modifying nanocellulose with TA and DA was adapted from Foo et al. (2019) and Hu et al. (2017) [4,38]. CNF and CNC aqueous suspensions (1%, *w*/*v*) were adjusted to pH 8 by addition of 1 M NaOH solution and then TA was added to the suspensions at the concentration of 1 mg/mL in the final solution. The mixture was stirred at 200 rpm for 6 h at room temperature to form NC-TA suspensions. Then, DA was added at the concentration of 40 mg/mL in the final solution. The resulted NC-TADA suspensions were stirred at 200× *g* rpm for 3 h, before being centrifuged for 10 min at 1000× *g* rpm. The supernatant was discarded, and the pellet was re-suspended in water, followed by another round of centrifugation. This step was repeated twice. Finally, the modified nanocellulose suspensions were collected and freeze-dried. CNF-TADA and CNC-TADA were produced with yields of 113.15% ± 4.45 and 118.33% ± 2.35 from CNF and CNC, respectively.

### 2.5. Characterization

#### 2.5.1. Attenuated Total Reflection Fourier-Transform Infrared Spectroscopy 

The Attenuated Total Reflection Fourier-Transform Infrared Spectroscopy (ATR-FT-IR) spectra were recorded using the Frontier™ MIR/FIR spectrometer from PerkinElmer in a scanning range of 550–4000 cm^−1^ for 16 scans at a spectral resolution of 4 cm^−1^. All analyses were performed in duplicate.

#### 2.5.2. Powder X-ray Diffraction 

Powder X-ray Diffraction (PXRD) analyses were performed on a Rigaku MiniFlex 600 diffractometer with Cu kα radiation, with a voltage of 40 kV and a current of 15 mA (3° ≤ 2θ ≥ 60°; step of 0.01 and speed rate of 3.0°/min). The Segal Crystallinity Index (CI, %) was calculated using the following equation:

(1) $$\mathrm{CI}\left(\%\right)=\frac{I_{t}-I_{a}}{I_{a}}\times 100$$

where *I_t_* is the total intensity of the (200) peak for cellulose I at 2θ = 22.5°, and *I_a_* is the amorphous intensity at 2θ = 18° for cellulose I [39,40]. All analyses were performed in duplicate.

#### 2.5.3. Zeta Potential Determination

Zeta potential (0.1%, *w*/*v* aqueous suspensions) was evaluated by dynamic light scattering (DLS) using a Malvern Instrument Zetasizer Nano ZSP (Malvern, UK). The measurements were made at room temperature (25 °C) using a folded capillary cell at a constant detection angle of 173°. The equipment was equipped with a 10 mW He-Ne laser with an emission wavelength of 633 nm. Data were acquired and analyzed using the Malvern Zetasizer v. 7.11 software. All measures were performed in triplicate.

#### 2.5.4. Microscopy Analysis

Morphology was evaluated by Scanning Electron Microscopy (SEM) on a Thermo Scientific™ Pro Scanning Electron Microscope. Prior to analysis, the samples were placed in observation stubs covered with double-sided adhesive carbon tape (NEM tape, Nisshin, Japan) and coated with Au/Pd (target SC510-314B from ANAME, S.L., Madrid, Spain) using a Sputter Coater (Polaron, Bad Schwalbach, Germany). All observations were performed in high vacuum with an acceleration voltage of 5 kV. The images presented are representative of the morphology of the sample.

Particles’ morphology was also evaluated by Transmission Electron Microscopy (TEM), where 10 µL of the samples were mounted on Formvar/carbon film-coated mesh nickel grids (Electron Microscopy Sciences, Hatfield, PA, USA) and left standing for 2 min. The liquid in excess was removed with filter paper and the grid was allowed to contact with a drop of uranyl acetate 2% (*w*/*v*) for 2 min. The liquid in excess was removed with filter paper. Visualization was carried out on a JEOL JEM 1400 TEM (Tokyo, Japan) at 120 kV. Images were digitally recorded using a CCD digital camera (Orious 1100W, Tokyo, Japan) at the HEMS/i3S of the University of Porto. 

### 2.6. Curcumin Loading

CNC as carrier was used for the preliminary curcumin:carrier ratio screening. The ratios tested were 1:2, 1:3, 1:5 and 1:10 (*w*/*w*). Calculated amounts of curcumin were dissolved in ethanol, and then CNC was added to the curcumin solution to achieve a final CNC concentration of 2% (*w*/*v*) in ethanol (70%, *v*/*v*), followed by continuous stirring at 250 rpm for 30 min. The curcumin-loaded particles were collected by centrifugation at 10,000 rpm for 10 min at room temperature. The unbound curcumin that remained in the ethanolic supernatant was analyzed by Ultraviolet-Visible (UV–Vis) Spectrophotometry using a BioTek Epoch 2 Microplate Reader (Winooski, VT, USA) and the pellet was freeze-dried after being washed with distilled water by centrifugation twice. Curcumin loading into CNF and functionalized materials (CNC-TEMPO, CNF-TEMPO, CNC-CTAB, CNF-CTAB, CNC-TADA and CNF-TADA) was performed as described above for CNC using the best performing curcumin:carrier ratio.

### 2.7. Curcumin Quantification

Curcumin was quantified by UV–Vis Spectrophotometry with detection at 425 nm. Five external standards of curcumin were analyzed (from 0.8 to 12.5 µg/mL) in duplicate and a curcumin calibration curve was constructed in ethanol (70%, *v*/*v*). The method presented good linearity with a correlation coefficient of 0.9999, and good precision results with coefficients of variation values lower than 5%.

### 2.8. Encapsulation Efficiency, Loading Capacity and Yield

The yield (%) was expressed as the ratio of the final freeze-dried mass obtained and the mass of the starting materials. The unbound curcumin (*Cur_unbound_*) that remained in the supernatant after curcumin loading was analyzed by UV–Vis Spectrophotometry. The encapsulation efficiency (*EE*%) and loading capacity (*LC*%) were calculated using the following equations:

(2) $$EE\left(\%\right)=\frac{Cur_{added}-Cur_{unbound}}{Cur_{added}}\times 100$$

(3) $$LC\left(\%\right)=\frac{Cur_{added}-Cur_{unbound}}{final\;mass}\times 100$$

### 2.9. Curcumin Release Profile

A simplified protocol that simulates the gastric and intestinal conditions was adapted from Gorbunova et al. (2018) and Valo et al. (2011) [41,42]. Fifty milligrams of each encapsulated particle were added to 20 mL of simulated gastric fluid (SGF) comprising a NaCl solution at 2 g/L adjusted to pH 2 by the addition of 1 M HCl. The experiment was performed at 37 °C for 2 h under agitation to simulate the digestion conditions at the stomach. Aliquots of 150 µL were withdrawn every hour and added to 350 µL of ethanol, and the extracted medium was replaced with fresh medium. The samples were centrifuged, and curcumin concentration was determined in the supernatant by UV–Vis Spectrophotometry. After 2 h in SGF, the samples were centrifuged and 20 mL of simulated intestinal fluid (SIF) comprising a KH_2_PO_4_ solution at 6.8 g/L adjusted to pH 7 by the addition of 1 M NaOH was added to the pellet. The experiment was performed at 37 °C for 6 h under agitation to simulate the digestion conditions of the intestine. Samples were withdrawn every hour as described above. The samples were centrifuged, and curcumin concentration was determined in the supernatant by UV–Vis Spectrophotometry. The percentage of curcumin released was determined and plotted over time. All experiments were performed in duplicate.

### 2.10. Cytotoxicity Evaluation

Cell Line Growth Conditions. Human colon carcinoma (Caco-2) cells were obtained from the European Collection of Authenticated Cell Cultures. Cells were cultured at 37 °C in a humidified atmosphere of 95% air and 5% CO_2_ as monolayers using Dulbecco’s Modified Eagle’s Medium (DMEM) with 4.5 g/L glucose, L-glutamine and no pyruvate (ThermoScientific, Massachusetts, USA), supplemented with 10% Fetal Bovine Serum (ThermoScientific, Massachusetts, USA), Penicillin-Streptomycin-Fungizone (1%, *v*/*v*) (ThermoScientific, Massachusetts, USA) and Non-Essential Amino Acids Solution (1%, *v*/*v*) (MEM NEAA, ThermoScientific, Massachusetts, USA). The cells were used between passages 28 and 32.

Cytotoxicity Assay. Cytotoxicity evaluation was performed according to the ISO 10993-5:2009 standard in Caco-2 cells [43]. The cells were grown to ca. 80% confluence, detached using TrypLE Express (ThermoScientific, Waltham, MA, USA) and seeded at 1 × 10^4^ cells/well into a 96-well microplate. After 24 h, the culture media was carefully removed and replaced with culture media supplemented with curcumin (concentrations between 0.01 and 0.2 mg/mL) or curcumin encapsulating particles (concentrations between 0.03 and 0.5 mg/mL). DMSO at 10% (*v*/*v*) in culture media was used as death control, and plain culture media was used as growth control. After 24 h of incubation, 10 µL of Presto Blue reagent (Thermo Fisher Scientific, Waltham, MA, USA) was added to each well and the plate was incubated for 2 h. After this period, fluorescence intensity measurements (Ex: 560 nm; Em: 590 nm) were performed in a BioTek Synergy H1 Microplate Reader (Winooski, VT, USA). All assays were performed in quadruplicate. Results were given in terms of percentage of the cell metabolism inhibition.

### 2.11. Statistical Analysis

All analyses were performed at least in duplicate and the results are reported as the mean values and standard deviations. As the data followed a normal distribution by the Shapiro–Wilk Test, one-way analysis of variance (ANOVA) followed by post-hoc Tukey’s test (*p* < 0.05) was conducted to determine the significant differences between mean values using the SPSS 28.0 Statistical Software Program (SPSS Inc. Chicago, IL, USA).

## 3. Results

### 3.1. Characterization of the Carrier Materials

#### 3.1.1. ATR-FT-IR Analysis

**Figure 1:**
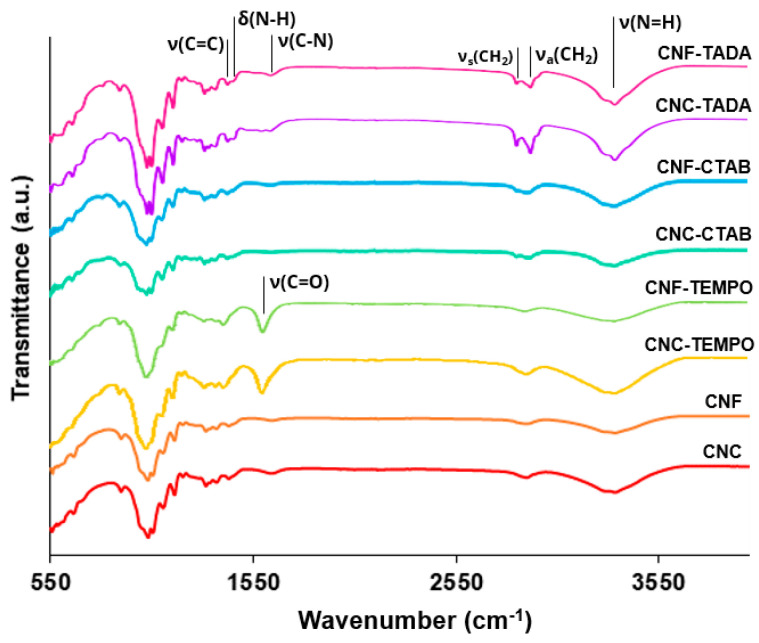
ATR-FT-IR spectra of the modified and unmodified cellulose nanocrystals (CNC) and cellulose nanofiber (CNF) materials. Nanocellulose modifications were performed with cetyltrimethylammonium bromide (CTAB), tannic acid and decylamine (TADA), and by TEMPO-mediated oxidation (TEMPO).

**Figure 2:**
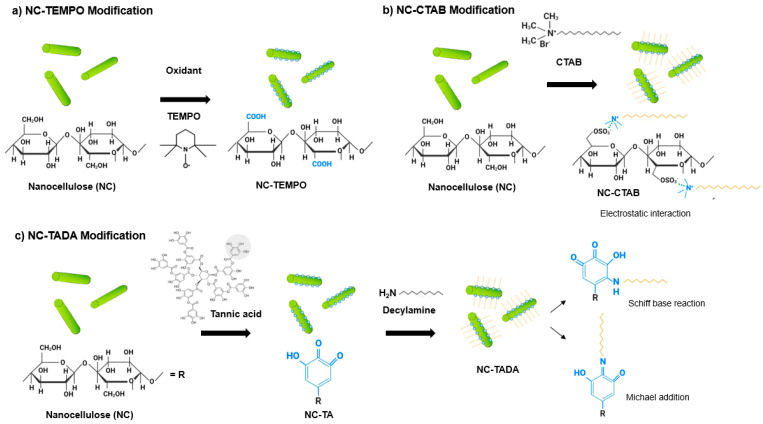
Illustration of (**a**) the nanocellulose-based product obtained by TEMPO-mediated oxidation; and the interaction of nanocellulose (NC) with (**b**) cetyltrimethylammonium bromide (CTAB) and (**c**) tannic acid and decylamine (TADA).

Commercial CNC, CNF and CNF-TEMPO, and prepared CNC-TEMPO, CNC-CTAB, CNF-CTAB, CNC-TADA and CNF-TADA were structurally characterized in terms of functional groups using ATR-FT-IR analysis (Figure 1). Vibrations related to cellulose modifications are identified in Figure 1. A schematic illustration of the molecular products obtained by the TEMPO-mediated oxidation of nanocellulose and modification with CTAB and TADA are represented in Figure 2.

Characteristic cellulose vibrations were found in all nanocellulose samples, namely at: 3200–3400 cm^−1^ (O-H stretching), 2900 cm^−1^ (C-H stretching), 1600–1640 cm^−1^ (O-H bending), 1430 cm^−1^ (CH_2_ bending), 1110 cm^−1^ (C-O-C stretching) and 900 cm^−1^ (C-H rocking), indicating that cellulose was preserved in all modification processes. Similar vibrations in the wavelength positions observed here have been reported in the literature for CNF [44] and CNC [45,46,47], but also for TEMPO-mediated [36,48,49], CTAB [28,35] and TADA [4,38] modified celluloses.

After oxidation, both CNC-TEMPO and CNF-TEMPO exhibited a new vibration at 1600 cm^−1^, corresponding to C=O stretching in carboxyl functional groups bound to a sodium ion (COONa) (Figure 2a). Similar vibrations have been reported in the literature for TEMPO mediated oxidized CNF [48,49] and CNC [36]. In general, characteristic vibrations on the spectra of CTAB modified nanocelluloses exhibited similar spectral properties with the unmodified CNC and CNF materials. Similar results have been reported in the literature by Qing et al. (2016) for CTAB modified CNC [35]. Only very small vibrations can be noticed at 2850 cm^−1^ and 2925 cm^−1^, also reported by Zainuddin et al. (2017) [28], which correspond to the symmetric and asymmetric CH_2_ stretching vibrations of the alkyl chain in CTAB. Modification with CTAB involves the physical adsorption of the surfactant molecules onto the surface of nanocellulose via electrostatic attraction of opposite charges (Figure 2b). Strong electrostatic interactions were due to the binding of the cationic charged head of CTAB with the negatively charged NC surfaces [28]. As the surfactant was non-covalently bound with nanocellulose, no major differences in the ATR-FT-IR spectra of modified and unmodified CNC and CNF were observed. 

For the TADA-modified samples, the presence of asymmetrical and symmetrical CH_2_ stretches from the C_10_ alkyl chain at 2850 cm^−1^ and 2925 cm^−1^ suggests the attachment of decylamine onto CNC and CNF [38]. Notable changes across the broad band in the 3200–3500 cm^−1^ region were also observed, with more prominent peaks at 3330 cm^−1^ and 3345 cm^−1^, which have been ascribed to N=H stretching [4]. Furthermore, new peaks at 1460 cm^−1^ corresponding to C=C aromatic stretching vibration, at 1470 cm^−1^ attributed to secondary N–H bending (indicating Michael addition occurred), and at 1650 cm^−1^ assigned to C-N stretching were detected, confirming the introduction of quaternary ammonium groups to CNC and CNF [4,38]. Moreover, the vibration intensity of the ether bond at 1055 cm^−1^ was stronger for all TADA-modified samples, which also indicates the attachment of long alkyl groups to the nanocellulose [28]. Similar vibrations in the wavelength positions observed here have been reported in the literature for TADA-modified nanocellulose [4,38]. The chemical structure of the tannic acid primer, containing catechol groups, and decylamine, which imparts hydrophobicity, is shown in Figure 2c. In step 1, tannic acid reacts with NC at pH 8, as under these conditions polyphenol oxidation and oligomerization is known to occur in the presence of available dissolved oxygen. This produces quinones that in the second step of the modification react with primary amine groups in decylamine via a Schiff-base reaction and/or Michael addition, as depicted in Figure 2c [38,50]. As strong covalent bonds are formed during this modification, clear and multiple changes in the ATR-FT-IR spectra of CNC and CNF were observed.

#### 3.1.2. PXRD Analysis

**Figure 3:**
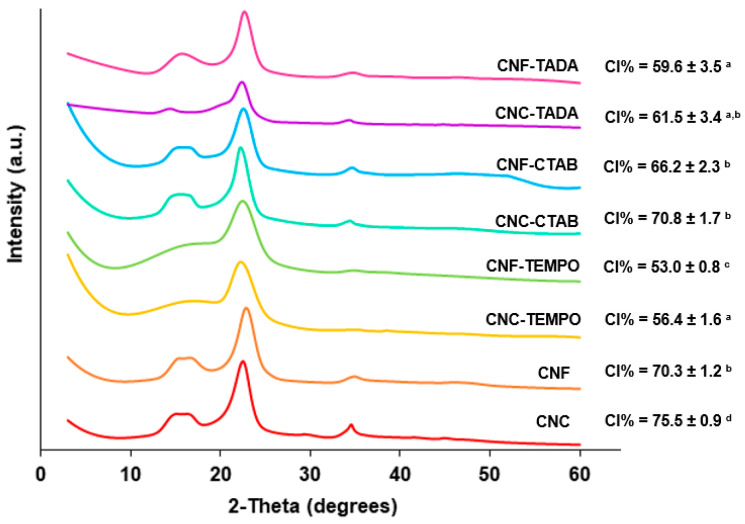
Powder X-ray Diffraction analyses of the modified and unmodified cellulose nanocrystals (CNC) and cellulose nanofiber (CNF) materials. Nanocellulose modifications have been performed with cetyltrimethylammonium bromide (CTAB), tannic acid and decylamine (TADA), and by TEMPO-mediated oxidation (TEMPO). (Averages followed by the same letters in the same column do not differ statistically by Tukey’s test (*p* > 0.05).

Commercial CNC, CNF and CNF-TEMPO, and prepared CNC-TEMPO, CNC-CTAB, CNF-CTAB, CNC-TADA and CNF-TADA were structurally characterized in terms of crystallographic structure using PXRD. The PXRD patterns and crystallinity indices are shown in Figure 3. 

All modified and unmodified nanocellulose samples exhibited cellulose characteristic reflections at 2θ = 15.0°/16.5°, 22.5° and 34.5°, corresponding to the (1-10)/(110), (200) and (004) planes of cellulose I, respectively [46,51], demonstrating that the cellulose structure was generally maintained. Nevertheless, TEMPO-mediated oxidation showed a statistically significant (*p* < 0.05) decrease in crystallinity (from 70–75% to 53–56%), which was supported by the less defined reflections at 2θ = 15.0°/16.5° and 34.5°, indicating an increase in the amorphous part. Similar results have been reported by Isogai et al. (2019) and Kim et al. (2021) [52,53]. 

For TADA-modified samples, cellulose peaks prevailed, but there was some broadening due to the modification, which was also observed by Hu et al. (2017) [38]. As expected, the crystallinity index significantly (*p* < 0.05) decreased after modification, for both CNC (from 75% to 61%) and CNF (from 70% to 59%). On the contrary, the attachment of CTAB to the surface of NC gave similar PXRD patterns to unmodified NC, implying that the underlying particle crystallinity was unchanged by the modification, as the surfactant was only electrostatically bound to nanocellulose. Even so, the crystallinity significantly (*p* < 0.05) decreased for CNC (from 75 to 70%) after CTAB modification. Decreases in CI of up to 5% from CNC to CNC-CTAB have also been obtained in the literature, which was been attributed to the disordered of the cellulose crystalline structure during modification, and the attachment of the bulky structure of a long alkyl chain of CTAB, which may increase the amorphous phase of the material [28,35]. 

#### 3.1.3. Zeta Potential

Dispersions with zeta potentials high in absolute value are electrically stabilized (repulsion exceeds attraction), while dispersions with low zeta potentials (attraction exceeds repulsion) tend to coagulate or flocculate [54]. According to Simões et al. (2020) and Ghalandari et al. (2015), a colloidal system is considered stable when showing ZP values above +30 mV or below −30 mV, thus meaning that the charge between particles (i.e., repulsion) is enough to prevent aggregation [55,56]. All nanocellulose suspensions exhibited negative zeta potential, with values ranging from −17 to −45 mV (Table 1). Both unmodified CNC (ZP = −45 mV) and CNF (ZP = −34 mV) originated stable suspensions, as ZP > ±30 mV. The extra negative charge of CNC indicates the attachment of negative sulfate groups (–OSO_3_^−^) on its surface, considering CNC is generally prepared by acid hydrolysis with H_2_SO_4_ [57]. TEMPO-mediated oxidized CNC and CNF also exhibited negative and stable suspensions (ZP = −31 to −34 mV), mostly due to the presence of negative carboxylate (COO−) groups at the surface of NC generated during the oxidative treatment. The same has been observed in works by Gamelas et al. (2015) and Zhou et al. (2018) [36,58]. Nanocellulose modification with CTAB and TADA, which targets the hydroxyl (OH-) groups on NC surface (to which negative sulfate groups are also attached in the case of CNC), led to a less electronegative zeta potential (*p* < 0.05), with results of −28 mV to −17 mV for NC-CTAB and −25 mV to −22 mV for NC-TADA. Similar results were reported by Jackson et al. (2011) for NC modification with CTAB 2 mM, and Foo et al. (2019) for TADA modification, demonstrating modified CNC structures with ZP ca. −30 mV and −15 mV, respectively [4,34].

### 3.2. Curcumin Binding Analysis

#### 3.2.1. Preliminary Curcumin:Carrier Ratio Screening 

An initial screening test was performed in order to optimize the curcumin:carrier ratio in the encapsulated systems. CNC was used as a carrier model for the screening. The ratios tested were 1:2, 1:3, 1:5, 1:10 and 1:15 (*w*/*w*) curcumin:carrier. The loading capacity and encapsulation efficiency results obtained are presented in Table 2.

The curcumin encapsulation efficiency was found to be a function of curcumin concentration between ratios 1:15 and 1:3 curcumin:CNC (*w*/*w*), with EE increasing significantly (*p* < 0.05) from 75 to 81% when the amount of added curcumin increased from 1:15 to 1:3 curcumin:CNC (*w*/*w*). A higher binding efficiency was also achieved when a higher amount of curcumin was added to nanocellulose materials in a work conducted by Zainuddin et al. (2017), who attributed this phenomenon to the increased number of active sites on curcumin molecular structure [28]. A higher curcumin:carrier ratio also allowed a higher loading capacity, with experimental values being consistent with the theoretical values for the ratios 1:10 to 1:3 (*w*/*w*). However, when an excessive amount of curcumin was added, a statistically significant (*p* < 0.05) decrease in EE and LC (in relation to the theoretical value) was observed, assessing from the results of the 1:2 curcumin:CNC (*w*/*w*) ratio. This can be attributed to a lack of ability from CNC to bind more curcumin molecules, suggesting that there is a limit to curcumin encapsulation by nanocellulose materials. From the data showed in Table 1, the maximum curcumin encapsulation efficiency (81.16% ± 0.21) occurred for the 1:3 curcumin:CNC (*w*/*w*) ratio, which corresponds to a loading capacity of ca. 25%. This curcumin:carrier ratio was used for further encapsulation studies.

#### 3.2.2. ATR-FT-IR Analysis of the Loaded Nanostructures

**Figure 4:**
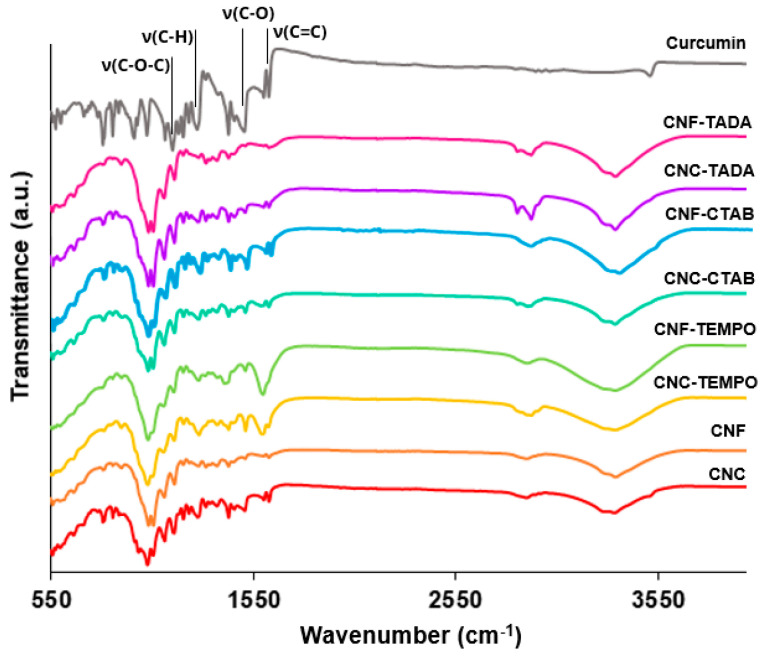
FT-IR spectra of the modified and unmodified cellulose nanocrystals (CNC) and cellulose nanofiber (CNF) systems encapsulating curcumin, as well as pure curcumin. Nanocellulose modifications have been performed with cetyltrimethylammonium bromide (CTAB), tannic acid and decylamine (TADA), and by TEMPO-mediated oxidation (TEMPO).

Figure 4 shows the ATR-FT-IR spectra of unmodified and modified nanocellulose (with CTAB, TADA and by TEMPO mediated oxidation) systems encapsulating curcumin, as well as the spectra of pure curcumin. The curcumin spectra showed characteristic vibrations at 1610 cm^−1^, 1500 cm^−1^, 1275 cm^−1^ and 1150 cm^−1^, which correspond to the stretching vibrations of the benzene ring, C-O stretching, C-H stretching and C–O–C stretching, respectively [59,60,61]. The same vibrations can be observed in the nanocellulose encapsulation systems, indicating the successful loading of curcumin onto the systems. Moreover, the curcumin characteristic peaks were not significantly shifted in curcumin loaded systems, revealing that there was no change in the chemical composition/structure of curcumin after the loading process. Nevertheless, the intensity of the bands on encapsulation systems was lower than in pure curcumin, suggesting the incorporation of curcumin into the delivery systems, as discussed by Iurciuc-Tincu et al. (2020) [62] 

#### 3.2.3. Curcumin Loading

Curcumin loading into modified CNC and CNF (with CTAB, TADA and by TEMPO-mediated oxidation) was examined and compared against the unmodified CNC and CNF. Yield, encapsulation efficiency, loading capacity and zeta potential results are presented in Table 3.

**Figure 5:**
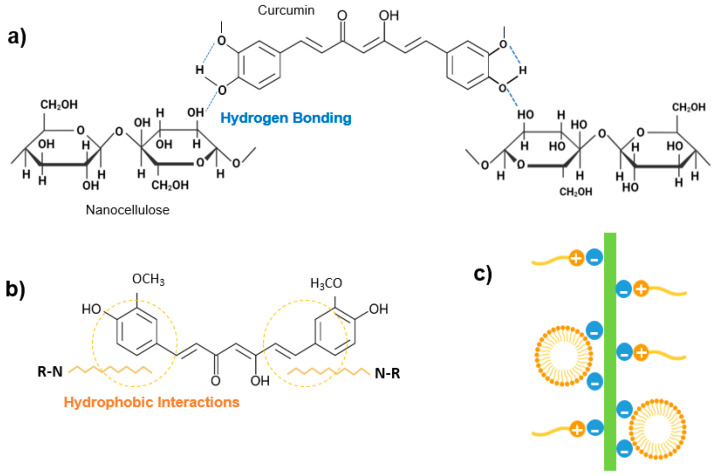
(**a**) Representation of hydrogen bonding between curcumin and nanocellulose structures; (**b**) Representation of hydrophobic interactions between curcumin and hydrophobic modified nanocellulose; (**c**) Schematic illustration of CTAB and nanocellulose interactions.

Unmodified CNC and CNF were able to bind ca. 85% of the added curcumin, with no significant differences (*p* > 0.05) found between the two systems. The oxygen of hydroxyl on the benzene ring of curcumin is a potential hydrogen donor and an important binding site for nanocellulose hydroxyl groups, and it is possible that an interaction occurred between curcumin and nanocellulose molecules through hydrogen bonding (Figure 5a) [59]. The large surface area of nanocellulose materials further potentiates the binding of curcumin molecules onto nanocellulose [11]. Lower curcumin EE of ca. 27% and 55% have been reported in the literature for unmodified nanocellulose materials, which may be due to the considerably lower amount of curcumin added in these studies, with curcumin:CNC ratios of 1:16 and 1:10 (w/w), respectively, as discussed in the ratio screening section [4,28]. 

Modifications of CNF with CTAB and by TEMPO mediated oxidation did not show significant differences (*p* > 0.05) in curcumin encapsulation from the unmodified CNF system, which might indicate that curcumin loading to CNF may be more related to fiber network entanglement and entrapment than actual molecule binding to CNF, as has been previously suggested by Kolakovic et al. [63]. On the other hand, when CNC has been modified by TEMPO-mediated oxidation, curcumin encapsulation significantly (*p* < 0.05) decreased to ca. 55%, indicating that the modification of hydroxyl groups to carboxyl groups decreased the ability of CNC to bind curcumin. 

On the contrary, when hydrophobic modifications were employed to CNC, statistically significant (*p* < 0.05) higher curcumin encapsulation efficiencies were observed. CNC-CTAB was able to bind ca. 90% of added curcumin, with a LC of ca. 25%, which matches the theoretical value. Similar results of curcumin EE of 80–96% have been reported in the literature for CNC-CTAB [28]. Due to its structure and hydrophobicity properties, curcumin may show the interaction between its benzene rings with the hydrophobic region of the modified CNC-CTAB (C-H moieties) via electrostatic and hydrophobic interactions (Figure 5b). These interactions in combination with the hydrogen bonding described earlier, are more effective in curcumin binding and encapsulation [4]. Furthermore, the adsorbed surfactant monomers can reorganize and induce hydrophobic interactions between the alkyl chains of the surfactant to form surfactant clusters, further favoring curcumin binding (Figure 5c) [28]. The binding capacity of nanocellulose significantly (*p* < 0.05) increased after TADA modification, achieving EE of ca. 99.8%, for both modified CNC and CNF. The increased level of hydrophobicity in NC-TADA favored the hydrophobic interactions between the hydrophobic phenolic moieties of curcumin and the long alkyl chain of DA (Figure 5b), resulting in remarkable curcumin-binding efficiencies. Furthermore, the excellent binding capacity of NC-TADA has been ascribed to the emergence of entanglement of NC-TADA and the formation of crosslinked-like networks, facilitating the binding of curcumin onto the surface of nanocellulose [4]. Curcumin EE ranging from 95 to 99% have also been reported in the literature for TADA modified CNC [4]. 

Generally, higher yields were observed for CNF systems (with CNF and CNF-TADA showing yields > 90%), indicating that the smaller size of CNC may have led to a loss of carrier particles during the encapsulation process (most likely during centrifugation). The fact that the loading capacity was also usually higher than the theoretical value of 25% for CNC systems also suggests that a loss of carrier materials may have occurred. However, for the CNC-CTAB-modified system this tendency was not observed, exhibiting a yield of 90.36% and a LC of 24.78%, which supports the success of this encapsulation approach.

Zeta potential results show that unmodified CNC encapsulating curcumin produced a very stable suspension (ZP < −30 mV), while all the other systems were moderately stable (ZP ca. −20 mV). The zeta potential became slightly less electronegative from the free nanocellulose materials (Table 1) to the curcumin-bound nanocellulose systems (Table 3). This increase has been observed in previous works and might be due to interactions between the charged NC surface groups and curcumin [35]. The fact that NC zeta potentials do not suffer major alterations provides an indication that no strong bonds are formed between carrier and curcumin, meaning that curcumin conserved its chemical structure and can potentially be released in active form from the nanocellulose materials. 

### 3.3. Curcumin Release Profile

**Figure 6:**
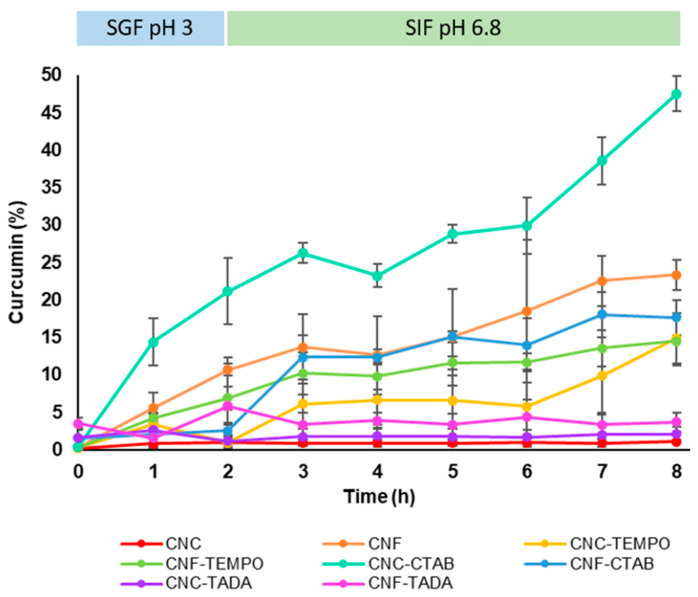
Curcumin release profile from modified and unmodified cellulose nanocrystals (CNC) and cellulose nanofiber (CNF) delivery systems in simulated gastric fluid (SGF, pH 3) and simulated intestinal fluid (SIF, pH 6.8). Nanocellulose modifications have been performed with cetyltrimethylammonium bromide (CTAB), tannic acid and decylamine (TADA), and by TEMPO-mediated oxidation (TEMPO).

The curcumin release profile from the nanocellulose delivery systems was studied in simulated gastric fluid for 2 h, followed by simulated intestinal fluid for 6 h, both at 37 °C, in order to simulate gastrointestinal conditions. Curcumin release profiles from the different nanocellulose systems (modified and unmodified) are shown in Figure 6. Unmodified CNC and CNF showed a slow release of curcumin, (18–23% after 8 h), while the NC TEMPO-mediated oxidized forms released ca. 15% over 8 h, which was a slight decrease in comparison to the unmodified NC structures. CNF-CTAB showed a similar release profile to unmodified CNF, with ca. 18% of curcumin being released after 8 h. On the other hand, CNC-CTAB showed an increased, but still sustained curcumin release capability, with ca. 48% of curcumin being released over 8 h, which was the highest amount of curcumin released in this study. This may be due to the different hydrophobicity of the carriers, allied to the non-covalent interactions between CNC and the surfactant, which may facilitate curcumin release from the carrier system, as opposed to NC-TADA. Studies available in the literature showed that CTAB modification enabled a sustained release of lipophilic bioactive compounds of 45–75% over 1–2 d in PBS [34,35]. The faster release of curcumin observed in the present study might be related to the simulated gastric conditions, in which the acidic medium might have helped in curcumin unbinding and faster release, as observed by Iurciuc-Tincu et al. (2020) [62]. Hydrophobic modification with TADA showed a small amount of curcumin released, since only ca. 2–4% curcumin was detected after 8 h, for both CNC-TADA and CNF-TADA. Despite the excellent encapsulation efficiencies obtained for this type of material, this system hindered the release of the bioactive compound in simulated biological fluids, and so it does not seem a promising curcumin delivery system for pharmaceutical and nutraceutical applications.

### 3.4. Cytotoxicity Evaluation

**Figure 7:**
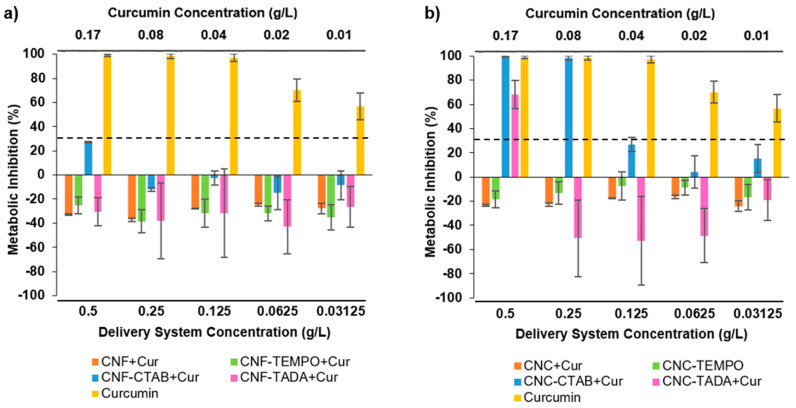
Impact of pure curcumin and modified and unmodified cellulose nanofiber (CNF) (**a**) and cellulose nanocrystal (CNC) (**b**) systems encapsulating curcumin upon Caco-2 cells metabolic activity. Nanocellulose modifications have been performed with cetyltrimethylammonium bromide (CTAB), tannic acid and decylamine (TADA), and by TEMPO-mediated oxidation (TEMPO). The dotted line represents the 30% cytotoxicity limit as defined by the ISO 10993-5:2.

Considering a potential gastrointestinal delivery of the developed nanocellulose delivery systems within pharmaceutical or nutraceutical applications, the cytotoxicity of the systems was assayed against the intestinal cell line Caco-2, in order to determine the maximum concentration that is not cytotoxic. The results are presented in Figure 7a,b for CNF and CNC systems (0.03–0.5 g/L), respectively, alongside pure curcumin (0.01–0.17 g/L, the same concentration that was present in the encapsulated systems). Considering a threshold for cell cytotoxicity of <30% metabolic inhibition (according to ISO 10993-5:2), curcumin proved to be cytotoxic to cells at all tested concentrations (metabolic inhibitions of 55–95%). Literature studies reported a concentration-dependent curcumin cytotoxicity, with cell viability being less than 80% for concentrations above 0.009 g/L (i.e., 25 µM) [64,65]. 

Delivery systems composed of CNF (Figure 7a) had no impact upon cells’ metabolic activity, with all CNF systems (unmodified and modified) presenting either no alterations or small promotions in the cell’s metabolism at all the concentrations tested. When considering the cytotoxicity results of CNC systems (Figure 7b), the scenario is somewhat different. Unmodified CNC and CNC- TEMPO also exhibited no significant impact upon cells metabolic activity, with no metabolic inhibition being observed. CTAB and TADA modifications, however, proved to be cytotoxic to cells depending on the concentration tested. Curcumin concentrations higher than 0.25 g/L for CNC-CTAB and 0.50 g/L for CNC-TADA led to significant reductions in cell metabolism, with metabolic inhibitions of 65–100%. Cytotoxicity concerns about the use of surfactants and amino compounds have arisen in the literature, and a previous study has shown that modified-NC with cationic surfactant CTAB can cause a significant reduction in cellular (fibroblasts 3T3) viability and proliferation [66]. However, despite the inhibitions observed in the present study, no cytotoxic effect was detected up to 0.125 g/L for CNC-CTAB and 0.25 g/L for CNC-TADA encapsulating curcumin, meaning that up to this concentration, the delivery system is not cytotoxic towards intestinal cells. It is interesting to note that curcumin encapsulation into CNF and CNC systems under safe concentrations was able to reduce curcumin cytotoxicity, as encapsulated curcumin did not exhibit metabolic inhibition as opposed to pure curcumin. The use of encapsulation techniques to overcome ingredient toxicity is a valuable approach to promote the applicability of interesting bioactive compounds [67,68]. 

### 3.5. Morphological Analysis

**Figure 8:**
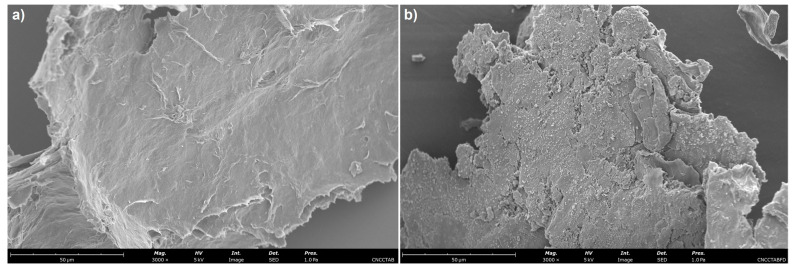
Scanning electron microscopy images of (**a**) free cellulose nanocrystals modified with cetyltrimethylammonium bromide (CNC-CTAB) and (**b**) CNC-CTAB encapsulating curcumin (3000×, 5 kV, 1.0 Pa, scale bar equals 50 µm).

**Figure 9:**
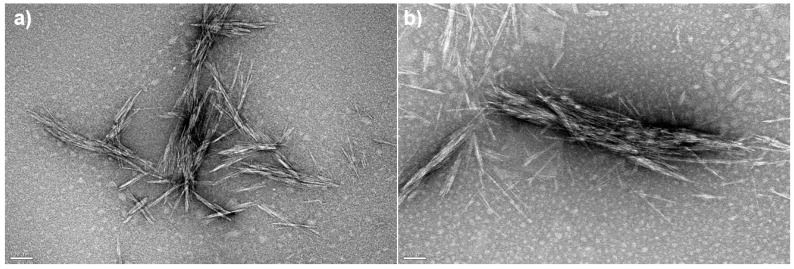
Transmission electron microscopy images of (**a**) free cellulose nanocrystals modified with cetyltrimethylammonium bromide (CNC-CTAB) and (**b**) CNC-CTAB encapsulating curcumin (100,000×, 120 kV, scale bar equals 100 nm).

The morphological properties of freeze-dried free CNC-CTAB and CNC-CTAB encapsulating curcumin, which proved to be the most promising delivery system in terms of encapsulation and release properties, were analyzed by SEM (Figure 8) and TEM (Figure 9). SEM images showed that the cellulosic structures form an aggregated polymeric laminar matrix during the freeze-drying process, as has been previously observed in the literature [69]. Small particles deposited on the surface of the encapsulating structures (Figure 8b) are likely to represent curcumin particles, suggesting the binding of curcumin onto CNC-CTAB. These particles were not observed in the free cellulose-based material (Figure 8a). The lamellar structures form multiple layers around the entrapped curcumin particles, in a similar morphology to the one observed by Kolakovic et al. (2012) for CNF entrapping the hydrophobic drugs itraconazole and indomethacin [70] On the other hand, TEM images were able to show individualized particles with a needle-like morphology and dimensions in the nanometric range. Similar morphologies have been reported in the literature for CNC modified with CTAB [28]. After curcumin encapsulation, the rod-like structure of CNC was not affected (Figure 9b), as previously described by Foo et al. (2019) [4]. However, the average diameter and length of the nanoparticles appears to have increased, suggesting the binding of curcumin onto CNC-CTAB.

## 4. Conclusions

In the present study, nanocellulose (cellulose nanocrystals and cellulose nanofibers) was tested as carrier for the delivery of curcumin, a model liposoluble compound. Hydrophobic modifications with the surfactant CTAB and tannic acid/decylamine (TADA) and modification by TEMPO-mediated oxidation, were also tested and compared with the unmodified nanocellulose structures. Structural characterizations confirmed the success of cellulose modifications, and the binding of curcumin onto the nanocellulose systems. The results showed that all systems encapsulated significant amounts of curcumin, ranging from 54 to 99%, depending on the structure used. We concluded that modification with CTAB and TADA resulted in effective curcumin encapsulation efficiencies of 90–99%. While NC-TADA was unable to release curcumin in simulated gastrointestinal fluids (2–4% released in 8 h), CNC-CTAB allowed for a curcumin sustained release of ca. 50% in 8 h, demonstrating to be the most promising delivery system. This system showed no cytotoxic effect towards intestinal cells up to 0.125 g/L. Furthermore, curcumin encapsulation allowed to reduce curcumin cytotoxicity, highlighting the potential of curcumin encapsulation into nanocellulose delivery systems. This work suggests nanocellulose as a promising sustainable delivery system, while cationic modification with CTAB proved to be a capable approach to alter its surface properties for more efficient binding and release of liposoluble compounds, increasing the range of curcumin concentrations that could be employed in pharmaceutical or nutraceutical applications. These findings also highlight the potential added-value applications of cellulose, the world’s most abundant natural polymer, which can be obtained from several renewable and sustainable sources, such as lignocellulosic biomass from industrial and agricultural wastes.

## Figures and Tables

**Table 1:** Zeta potential results of modified and unmodified CNC and CNF.

Sample	Zeta Potential (mV)
CNC	−45.03 ± 0.79 ^a^
CNF	−34.20 ± 1.03 ^b^
CNC-TEMPO	−34.37 ± 1.04 ^b^
CNF-TEMPO	−30.90 ± 1.68 ^c^
CNC-CTAB	−27.97 ± 2.01 ^c^
CNF-CTAB	−17.47 ± 2.25 ^d^
CNC-TADA	−22.03 ± 0.84 ^d,e^
CNF-TADA	−24.90 ± 1.37 ^e^

Legend: CNC—cellulose nanocrystals; CNF—cellulose nanofibers; TEMPO—oxidized by 2,2,6,6-tetramethylpiperidine 1-oxyl; CTAB—modified with cetyltrimethylammonium bromide; TADA—modified with tannic acid and decylamine. ^a–e^ Means within the same column, labeled with the same letter do not differ statistically by Tukey’s test (*p* > 0.05).

**Table 2:** Curcumin encapsulation efficiency (EE) and loading capacity (LC) (both experimental and theoric) of nanocellulose systems at different curcumin to nanocellulose ratios.

Ratio Curcumin:CNC	EE (%)	LC (%)	Theoric LC (%)
1:2	56.64 ± 0.76 ^a^	26.90 ± 0.83 ^a^	33.33
1:3	81.26 ± 0.21 ^b^	24.57 ± 0.30 ^b^	25.00
1:5	79.46 ± 0.25 ^c^	16.04 ± 0.42 ^c^	16.67
1:10	78.31 ± 0.19 ^d^	8.82 ± 0.23 ^d^	9.09
1:15	74.97 ± 0.83 ^e^	3.13 ± 0.86 ^e^	6.25

Legend: CNC—cellulose nanocrystals; EE—encapsulation efficiency; LC—loading capacity. The theoretical LC (%) was expressed as the ratio of the amount of curcumin added and the mass of the starting materials (cellulose-based carrier material and curcumin). ^a–e^ Means within the same column, labeled with the same letter do not differ statistically by Tukey’s test (*p* > 0.05).

**Table 3:** Yield, encapsulation efficiency (EE), loading capacity (LC) and zeta potential (ZP) of modified and unmodified nanocellulose systems encapsulating curcumin.

Delivery System	Yield (%)	EE (%)	LC (%)	ZP (mV)
CNC	68.01 ± 3.22 ^a^	84.29 ± 3.06 ^a^	31.00 ± 3.41 ^a^	−49.83 ± 2.75
CNF	91.22 ± 3.64 ^b^	84.81 ± 4.79 ^a^	24.72 ± 2.12 ^b^	−19.67 ± 5.52
CNC-TEMPO	43.33 ± 4.01 ^c^	54.52 ± 3.29 ^b^	31.50 ± 3.45 ^a^	−26.13 ± 0.58
CNF-TEMPO	68.90 ± 2.73 ^a^	85.39 ± 0.28 ^a^	25.41 ± 2.70 ^b^	−25.13 ± 0.90
CNC-CTAB	90.36 ± 3.24 ^b,d^	90.23 ± 1.58 ^c^	24.78 ± 3.47 ^b^	−21.97 ± 1.86
CNF-CTAB	68.95 ± 3.40 ^a^	81.27 ± 2.97 ^a^	26.98 ± 3.29 ^b^	−15.37 ± 1.18
CNC-TADA	83.06 ± 2.89 ^d^	99.85 ± 0.23 ^d^	30.10 ± 1.06 ^a^	−20.90 ± 2.43
CNF-TADA	93.25 ± 2.16 ^b^	99.84 ± 0.31 ^d^	26.80 ± 2.29 ^b^	−21.91 ± 1.96

Legend: CNC—cellulose nanocrystals; CNF—cellulose nanofibers; TEMPO—oxidized by 2,2,6,6-tetramethylpiperidine 1-oxyl; CTAB—modified with cetyltrimethylammonium bromide; TADA—modified with tannic acid and decylamine. ^a–d^—Means within the same column, labeled with the same letter do not differ statistically by Tukey’s test (*p* > 0.05).

## Data Availability

The datasets used and/or analyzed during the current study are available from the corresponding author on reasonable request.
